# Mechanism of cotton resistance to abiotic stress, and recent research advances in the osmoregulation related genes

**DOI:** 10.3389/fpls.2022.972635

**Published:** 2022-08-17

**Authors:** Shah Saud, Lichen Wang

**Affiliations:** College of Life Sciences, Linyi University, Linyi, China

**Keywords:** cotton, resistance, resource exploration, molecular marker, gene cloning

## Abstract

Abiotic stress is an important factor affecting the normal growth and development of plants and crop yield. To reduce the impact of abiotic adversity on cotton growth and development, the material basis of cotton resistance and its physiological functions are analyzed at the molecular level. At the same time, the use of genetic engineering methods to recombine resistance genes has become a hot spot in cotton resistance research. This paper provides an overviews of the resistance mechanism of cotton against the threat of non-biological adversity, as well as the research progress of osmoregulation-related genes, protein-acting genes, and transcription regulatory factor genes in recent years, and outlines the explored gene resources in cotton resistance genetic engineering, with the aim to provide ideas and reference bases for future research on cotton resistance.

## Introduction

Cotton is an important cash crop and occupies an important position in the industrial and agricultural economy of the country (Abdelraheem et al., 2019). Cotton fibers, seeds and stalks are used extensively in textile, food and feed processing, and papermaking. Currently, China is the world’s largest producer and consumer of raw cotton (Khan et al., 2020). However the shortage of water resources, ecological problems such as soil salinity, population expansion, environmental pollution and other human activities have made the phenomenon of land disputes over food and cotton are becoming more serious around the world (Solomon et al., 2007; Dos Reis et al., 2012; Ullah et al., 2017). The cultivation of cotton in china has paid more attention has been paid to improving yield and quality traits and developing disease and insect resistance (Cun et al., 2002; Davis et al., 2006). However, there is very little research on resistance and adaptability to abiotic adversity.

**FIGURE 1:**
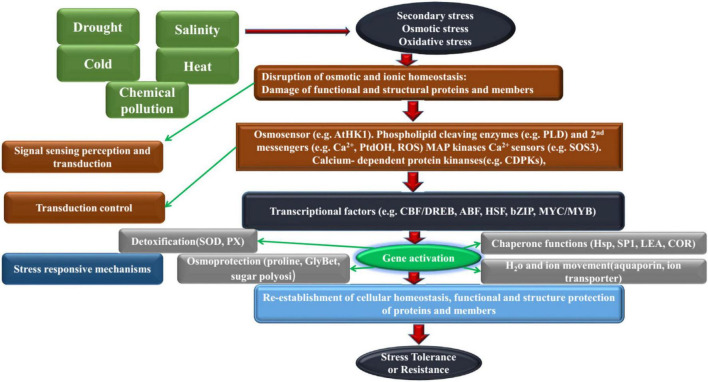
The complexity of plant response to adversity stress.

Abiotic stress mainly includes environmental factors that cause adverse effects such as extreme temperature, drought, salinity and alkali and low temperature usually cause a series of physiological and biochemical reactions in plants to adapt the adverse environment. These factors influence the development and growth of plants in addition to their morphological, physiological, biochemical and molecular processes (Liu et al., 2014; Zhang L. et al., 2014; Zhang et al., 2021). Plant responses to abiotic stress have been the subject of numerous studies worldwide. Initially, they were carried out on model plants and later on several important crops such as rice, corn, soybean, coffee and others (Ashraf, 2002; Gorham et al., 2010; Adams, 2011; Tiwari et al., 2013; Campbell et al., 2017). In order to adapt the environment and survive, plants have formed a series of resistance or tolerance to adverse environmental stresses during their long-term evolution resistance of plants. The resistance of plants is not just the result of a single mechanism. It is a very complex process (as shown in Figure 1). With the development of molecular biology, it has enabled people to understand the mechanism of plant resistance to stress at the molecular level of genetic composition, expression regulation, and signal transduction, especially in the molecular response of plants caused by abiotic stress. They will have severe impacts throughout the cotton growth and development stage, including a range of changes in plant morphology, physiology, quality and yield (Pei et al., 2020; Abro et al., 2022). Therefore, studying the adaptability of cotton to abiotic adversity and cultivating new varieties of resistant cotton are of great practical significance for cotton production.

The response pathway of cotton to abiotic stress can be roughly divided into four stages, ① the perception phase, the cotton senses the signal of abiotic adversity; ② the abiotic adversity signal is transmitted after the transmission stage; the reaction stage; the signal initiates a series of downstream molecular reactions ④ Expression stage; induces the expression of resistance-associated genes after going through the above four phases, cotton shows a specific response and resistance to the threat of non-biological adversity (Li et al., 2020). This paper summarizes, the research progress of cotton resistance response, development, utilization and molecular breeding of cotton resistant germplasm resources under abiotic stress, and proposed the development direction of cotton resistant breeding research in the future.

## Effects of non-living stress on cotton growth and development

Non-biotic stress mainly includes drought, salinity-alkaline, polar temperature, soil nutrient deficiency and other unhealthy environmental conditions (Yao et al., 2011; Dongdong et al., 2016; He et al., 2016; Liang et al., 2016; Gao et al., 2018). The normal growth and development of cotton was inhibited by severe non-living stress, resulting in cotton plant yield reduction or even death.

### Effects of abiotic stress on cotton growth and development cotton

**FIGURE 2:**
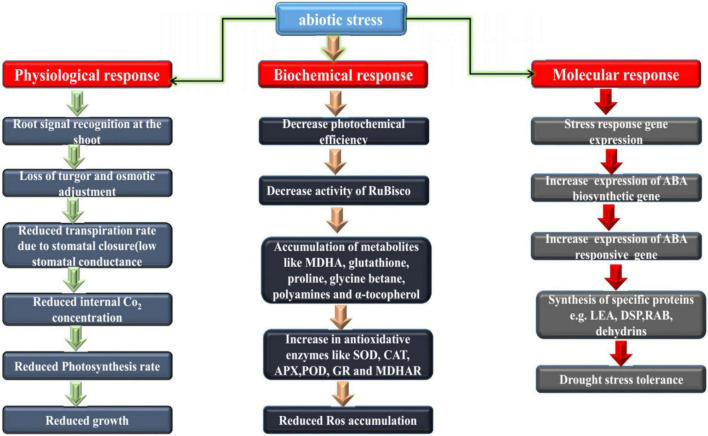
Physiological, biochemical and molecular basis of drought resistance in plants. Both major and minor changes that occur downstream of the transcriptional regulatory network.

Loss of crop production due to drought ranked first among all non-plant related pressures, and second among crop-related pressures. Drought can cause severe dehydration of plant cells; the normal cell membrane structure is destroyed, resulting in excessive closure of gas pores, which affects the absorption and uptake of CO_2_ and the decrease in photosynthesis in Figure 2. Chaves and Pereira (2003) and Onaga and Wydra (2016) investigated the influence of water stress on cotton seed germination, suggesting that cotton seeds under different drought stress were associated with decreases in water potential, germination rate, index, seedling height, root length, root-to stem ratio, dry weight and in associated with the fresh weight of young seedlings all decreased to varying degrees (Pettigrew, 2001; Fahad et al., 2017). Studying the growth and development of cotton in Jiangsu Province under drought conditions (Zhao et al., 2020), it was found that cotton growth was initially inhibited under drought conditions, and the plant height and leaf emergence rates were significantly slowed (Gao et al., 2020) found that when cotton was subjected to drought stress at different growth stages, its growth, development and yield were affected to some extent, and the influence from largest to smallest was the flower-boll, bud, maturity and seedling stage (Ullah et al., 2017; Ayele et al., 2020). The results show that drought resistant germplasm materials can effectively reduce water loss by increasing atmospheric pore resistance and reducing evapotranspiration intensity, thereby ensuring photochemical efficiency. Under drought conditions, root activity and proline accumulation of drought-resistant materials increased significantly (Liu et al., 2008).

### Effects of salt and alkali on cotton growth and development

Salt damage is one of the most important impairments in agricultural production. The concentration of salt in an appropriate amount can be used as a nutrient component to promote the normal growth of cotton. However, high salt concentration leads to early leaf maturation or early decay, resulting in a reduction in cotton yield (Pettigrew, 2004; Ennahli and Earl, 2005; Davis et al., 2014; Zhang X. et al., 2014). Under normal conditions, salt stress occurs when soil salinity is between 0.20 and 0.25%. Ion toxicity caused by salt stress, osmotic stress and oxygenation stress cause cotton to become infected or even die. High soil salinity leads to water absorption, swelling and slow germination of seeds, low germination efficiency (Stroup et al., 2003). The lack of water in root system caused infiltration stress, which led to dehydration of the cells and loss of turgor pressure, which affected the physiological function of cotton. The higher salt concentration, the more severe osmotic stress (Zhang X. et al., 2014). At the same time, too many salt ions in the soil also create ion competition and inhibit the uptake of other ionites by plants. It was found that NaCL stress significantly reduced the levels of Ca^2+^ K^+^, Mg^2+^, P^3^, and Mn^4+^ in the leaves and roots of cotton seedlings, leading to nutritional imbalance of cotton (Du et al., 2017; Hu et al., 2017; Tränkner et al., 2018; Zhao et al., 2020). Plants have adapted to salt stress environments and evolved an adaptation mechanism. It is mainly divided into salt ion injury regulation and joint osmosis (Zhang L. et al., 2014; Iqbal et al., 2020a). The high content of K^+^/Na^+^ and Ca^2+^/Na^+^ in the fabric is an important indicator of the salt tolerance of cotton. The root system of salt-tolerant cultivar had some amount of Na^+^, and the K^+^/Na^+^ in the body was significantly higher than that of the less salt-tolerant cultivar (Marschner, 2012; Oosterhuis et al., 2014) reported that high salt concentration enriched, the leaves become more salt-sensitive varieties had more Cl^–^ while the leaves of salt-tolerant varieties contained more K^+^, Ca^2+^, and K^+^/Na^+^. Armengaud et al. (2009) showed that selective potassium transport in the root could sustain the higher K^+^/Na^+^ content in the leaves, and this selective transport was an important feature of salt tolerance of cotton (Liu et al., 2008; Iqbal et al., 2020b; Zhao et al., 2020). Betaine is an important permeable modulating substance. When plants are subjected to environmental stress, betaine not only accumulates in cells to reduce osmotic potential, but also acts as a sort of preservative to maintain structure and maintaining integrity of macromolecules. At the same time, the membrane lipid peroxidation clearance system with high activity is very important for the stability of membrane structure and energy to maintain its normal physiological function (Fahad and Bano, 2012; Fahad et al., 2013), the activity of membrane lipid peroxidation removal system greatly increased and glutathione reducing enzyme activity by 55–101% compared to the photography increased 38–72%, and monosintin α content increased by 212∼320%, but the above indices did not change or decreased in salt-sensitive varieties.

### Influence of temperature change on cotton growth and development

The change in pole temperature caused growth, breeding and injury of cotton. Polar temperature changes include low and high temperature stress, and low temperature stress is divided into cold damage and frost damage, which slow or stop plant activity (Nievola et al., 2017).

After the cotton suffered low temperature injury, and withered the whole plant or part of the dead. The damage degree of cotton was closely related to the impact of low temperature on cotton at the seedling stage (Zakaria, 2017), when the degree of frostbite was more than 15%, the growing period of cotton was delayed, and the yield and quality of cotton were affected (Fahad et al., 2014a,b). Under low temperature strips, the germination rate of cotton seeds slowed to the point of rotting. Even when the seeds were germinated, the growth rate of bud length and weight was significantly reduced, so the degree of firmness of cotton seedlings was significantly affected. Low temperature and low light stress leads to a decrease in photochemical activity inhibition of photoelectron transfer, and disruption of excitation energy distribution and transfer between PSI and PSII (Abdurakhmonov et al., 2010; Rantala and Tikkanen, 2018). The increase in nitrate reductase activity is an indication of the cold resistance of cotton. Under low temperature treatment, nitrate reductase activity of low temperature tolerant cultivars increased, and plant mass contained increased nitrogen content to withstand unfavorable low temperature environment (Iqbal et al., 2020b).

Cotton is native to low latitudes in the tropics, but due to the long period of human selection and domestication, its ability to withstand high temperature is not strong. High temperature stress can affect cotton seed germination and inhibit root line growth and plant growth. The optimal temperature ranges for germination and growth of cotton seeds were 28°–30°C, and the optimal temperature ranges for root growth were 30^°^–35°C and 22^°^–27°C (ii). High temperature stress (40^°^C during the day and 32^°^ at night) reduced the rate of seed germination (Singh et al., 2007), impaired the uptake of water and nutrients in the root system, disrupted the formation and transport of pathogens and impaired the spread of the root system, as a result of which the root system led to a flatland formation. Tap and lateral roots are thin and poorly developed even under optimal water nutrient conditions (Ziska, 2000; Reddy et al., 2011). High temperature accelerate plant growth, shortens the boll growth period and carbon assimilation time, thereby reducing dry material accumulation, resulting in smaller bolls, lower yield, and worse cotton fiber quality (Thorp et al., 2020). Studies show that the relatively high temperature at night also affects the quantity and quality of buds and seed pods. In the reproductive growth phase, pollen germination is extremely sensitive to high temperature, and the optimum temperature is 28^°^C, while the pollen tube growth rate is linearly correlated with temperature below 37^°^C. At high temperatures, pollen viability, pollen germination rate and number of pollen grains at the column head decreased significantly, leading in pollen abortion (YunfeiJiang et al., 2017). The lowest, highest and optimal flower bud and tube growth temperature were determined by examining the flower bud and tube growth temperature of different cotton varieties (Kakani et al., 2005; Liu et al., 2006), at the same time, under high temperature stress, all organisms produce heat shock response (heat shock protein) to protect cells and organisms from serious damage (Craita, 2022). To regain normal cell viability and physiological viability. Therefore, the heat resistance of plants could be improved by increasing the surface level of heat stimulated protein (heat shock protein, HSPS).

### Effects of soil nutrient stress on cotton growth and development

Nitrogen, phosphorus and potassium are three essential elements for plant growth, which play irreplaceable roles in plant life and have a major impact on cotton yield and quality. Under potassium deficiency has a major impact on yield and fiber quality (Yibati et al., 2022), potassium deficiency significantly inhibited the elongation and lateral rooting of the cotton root system. Potassium deficiency can inhibit the elongation and thickening of the main cotton stem, affecting the vegetative growth of cotton. It also inhibited the production of albumin content, chlorophyllin content and effective rate of photosynthesis in functional leaves of cotton main stem (Fontana et al., 2020). Cotton lack of potassium for a long time, they are prone to red petiole rot, so nutrition and reproductive growth will blocked, the growth potential of cotton plant will be weakened, early decline and other hair diseases (Zhang L. et al., 2014). Cotton cultivars with high potassium efficiency had a strong ability to transfer potassium to shoot, thereby preserving the physiological energy of chloroplasts in cotton leaves. With the decrease of potassium concentration, the number of root hairs in potassium efficient varieties increased, and site formation was closer to the root tip (Shahzad et al., 2019; Bamagoos et al., 2021). Xylem conduit differentiation has improved and tissue transport capability has been enhanced. With the increase in root-shoot ratio and length, the indexes of root absorption energy and root viability increased significantly, while the content of malondialdehyde (MDA) was lower than that of the low-effect varieties. Therefore, the stability of membrane system of the high-effect varieties was better and the resistance of the high-effect varieties was stronger under low potassium stress (Yang et al., 2021).

There are many adaptive changes in plants under low phosphorus stress, including root morphology, root system secretion, membrane and internal phosphorus transport system, etc. Phosphorus deficiency has the greatest impact on cotton growth and yield, resulting in delayed plant growth and development smaller leaves, thinner, weaker brittle stems, shorter than normal plants, lower root growth, delayed boll setting and maturation little boll, low yield, poor fiber quality (Hodge, 2004; Grossman and Rice, 2012). Under low phosphorus stress, main root growth was limited, but lateral root density and length were increased, and root hair the number and length were also increased (Linkohr et al., 2002). The plasma membrane is the decisive factor for phosphorus uptake efficiency in plants, and the plasma membrane of root cells first link to main transport phosphorus inverse concentration. The migration of mechanized phosphorus across the cell membrane into the cell is regulated by the same or different families of phosphorus transporting albumen, which can be subdivided into Pht1, Pht2, and Pht3, families according to sequential similarity. They are located on the membrane of the root system, the inner membrane of the plastids and the membrane of the mitochondria (Ma et al., 2001; He C. et al., 2005; He Z. et al., 2005). They are responsible for the uptake of soluble phosphorus from the soil solution, the transfer of inorganic phosphorus from cytoplasm to plastid, and the exchange of linear phosphorus (Hodge, 2004).

The amount of nitrogen fertilizer applied to our country’s agricultural fields is increasing year by year, but the loss of nitrogen fertilizer is becoming more and more serious. Nitrogen uptake efficiency and nitrogen use efficiency (NUE) isthe main cause of difference in nitrogen efficiency (Fahad et al., 2013). Under the low nitrogen conditions, the difference in nitrogen efficiency was mainly due to the difference in nitrogen utilization rate, while under the high nitrogen conditions, the nitrogen absorption efficiency was the main determinant. Under nitrogen stress, cotton root lines could absorb nitrogen in lower concentration by increasing the percentage of active absorption area, to increase growth and development of root lines and plants. The area of active uptake and harvesting area as well as the specific surface area were the main reasons for difference between varieties with different nitrogen efficiencies. When nitrogen is applied, HCO^–^_3_ or OH^–^ was released into the rhizosphere and pH in the rhizosphere increased. In the absence of nitrogen, the morphological and physiological changes of cotton root could increase the utilization rate of nutrients by secreting protons, organic acids or increasing the activity of some enzymes (Fahad et al., 2013). Nitrogen efficient varieties adapted to lower nitrogen concentration and increased nutrient availability by increasing root shoot ratio, root density and root length. The results showed that the distribution ratio of nitrogen content in each organ of cotton from large to small leaf, stem and root, indicating that the cotton plant transported more nitrogen to the upper part of the soil, promoted photosynthesis (Niu et al., 2021).

## Research progress on cotton stress resistance breeding

In recent years, the shortage of global water resources, soil salinity, frequent extreme weather conditions and so on, the demand for cotton side-effect resistance is getting higher and higher. Cotton breeding not only focuses on improving yield, quality and resistance to diseases and insects, but also pays increased attention to resistance to abiotic stress and efficient use of soil nutrients (Diouf et al., 2017; Abdelraheem et al., 2021). Explore the optimal resistance germplasm resources of cotton, separate and calibrate the target characteristic genes/QTL of resistance/resistance to different adversity, clone and functionally clarify the candidate genes related to resistance (Wang et al., 2003), carry out research on the mechanism of interaction between cotton and different adversity, molecular design and creation of new materials for resistant cotton, and lay the foundation for resistant breeding of cotton in Figure 1.

### Exploration of cotton resistant and superior heterologous materials

Research on cotton resistance started late, however has progressed rapidly in recent years. At home and abroad, the collection, assessment and verification of cotton resistant germplasm resources on the non-biotic resistance of cotton (Nazeer et al., 2014; Yin et al., 2020) such as salt resistance, drought resistance, polar temperature tolerance, and soil trophic stress, and a number of important resistant germplasm resources were discovered. It is an important raw material for the resistance and breeding of cotton.

#### Exploration of drought-resistant and salt-resistant cotton resources

Cotton is recognized a salt and drought-resistant crop, and is the “pioneer crop” for desalination beach soil. It is an important source for increasing cotton acreage in the future to alleviate the conflict between grain and cotton and to utilize the reclaimed land. Selecting and breeding to salt-tolerant and drought-resistant cotton varieties to improve the adaptability of cotton to a salt-tolerant environment is one of the important ways to reduce the damage of salt-tolerant soils and to develop and utilize salt-tolerant land resources such as coastal beach (Sun et al., 2021; Zhang et al., 2022). The selection of drought-resistant and salt-tolerant raw materials is the basis for achieving this goal (Li et al., 2019). Seedling stage drought method to simulate drought environment to identify the drought resistance of 76 seedlings of cotton cultivars. 2 drought tolerant varieties (1193B and 18 short-season) and 5 sensitive varieties were selected (9811-36, 9810-3, 21025-2, China Cotton Institute 45 and 21021-8 (Hou et al., 2018). The above-mentioned varieties were further investigated for salt resistance, and it was found that low-NaCL concentration promotes seed germination while high concentration inhibits seed germination by evaluating salt resistance varieties and salt-sensitive varieties (Sun et al., 2018). The ship also screened three salt-resistant strains such as Salt 1032, 1129, and 2018. At the same time, it was found that the salt resistance of the new cotton variety Zhongmian Institute 76 significantly exceeded that of the comparison.

#### Exploration of cotton resources resistant to extreme temperature

Conductivity is an important indicator of cell membrane stability and fluidity and identifies the cold resistance of seedlings. The results showed that under low temperature stress, young cotton seedling electrical conductivity enzyme activity of peroxidants and soluble sugars increased, and root activity decreased, while young cotton seedling electrical conductivity and root activity decreased less in low temperature resistant varieties (Anastasia et al., 2010; Lin et al., 2021). The content of active and soluble sugars increased significantly. Thermal cell stability, proline, soluble sugar and photosynthetic rate of functional leaves of cotton main stem can be used as an important indicator for screening of cotton resources with high temperature tolerance (Hasanuzzaman et al., 2013). Currently, there is not much research on choosing high temperature resistant material for cotton pole. The results showed that anther development and pollen fertility of different cotton materials differed greatly in response to high temperature stress. The anther development and pollen fertility of high temperature resistant cotton materials were all normal, while the anther of sensitive cotton lines were dry, unruptured, and showed no male fertility (Bita and Gerats, 2013; Zahid et al., 2016). A group of raw materials with different tolerance to high temperature stress as sorted out. Using the above materials, the study on changes in physiological indicators of various high temperature tolerance cotton lines, such as SOD, POD, CAT, and MDA, was carried out. It laid the foundation for advancing separator research and breeding new cotton varieties in an environment of non-stop high temperature.

#### Exploitation of cotton resources with high effective nutrients

There were significant differences in nutrient uptake and availability between different cotton lines. Selecting the screening index of the combination and digging up nutrition and beneficial strains can improve the absorption and utilization efficiency of nutrients, improve the utilization rate of seed resources, reduce resources, protect the environment, and achieve sustainable agricultural development. Zhang et al. (2018) showed that the optimal seedling age for screening low potassium tolerance genotypes in cotton was 5 leaves. Echer et al. (2020) screened cotton varieties with high potassium efficiency genotypes under sand culture condition, and proposed that the potassium utilization index, potassium uptake and biomass were used as screening criteria for high potassium nutrition at the cotton seedling stage (Chattha et al., 2022). Five potassium efficient cultivars (602, Xinluzao 6, Jiaomian, 18-3 and Xinhai 13) and three potassium inefficient cultivars (Shi K7, Xinluzao 10, and Xinhai L4) were selected by the culture of the broth and two levels of potassium deficiency and appropriate potassium treatment, 86 cotton cultivars of different pedigree were screened for the potassium efficient basal type, and 4 varieties with high potassium efficiency and 2 varieties with low potassium efficiency were obtained. It is suggested that cotton lint yield and the potential to increase yield during potassium application are the ultimate indexes to judge whether cotton has high potassium efficiency. Through a hydroponics experiment (Zhang et al., 2021), two of high and low phosphorus treatments were established and 32 cotton varieties with high phosphorus genotypes were screened at seedling and bud stages from self-breeding or introduction in Xinjiang. Three phosphorus efficient and inefficient varieties were obtained. The results showed that the coefficient of variation of relative dry matter weight, relative phosphorus uptake and relative total plant phosphorus content were higher than those of other traits, that could be used as screening indices for phosphorus use efficiency at seedling and bud stages of cotton (Li et al., 2019), studied cotton varieties resistant to withered and verticillium wilt by vermiculite and irrigation with nutrient solution irrigation, and obtained two categories of varieties resistant to low phosphorus and sensitive to low phosphorus. The results showed that there were significant differences in cotton seedling dry matter, aerial fresh weight, total leaf area, chlorophyllin content, and dry matter weight the aboveground and phosphorus utilization rates between different phosphorus concentration and different varieties. It can be used as an index of assessment of low phosphorus tolerance of cotton at the seedling stage.

By determining the height, dry weight and total nitrogen content of cotton plants (Song et al., 2017), the seedling stages screening of nitrogen use efficiency of 48 cotton varieties self-bred or introduced in Xinjiang was completed to by 4 nitrogen-efficient varieties (602, Xinlu Early l2, Xinhai L2, and Xinlu Early 8) and 5 nitrogen-inefficient strains (Xinhai #. 14, Xinhai #. 2 and #. 1 Battery 1, Xinlu Early #. 2O and #. 18-3). Relative dry weight has a high degree of sensitivity to low nitrogen resistance of different cotton species, and correlates well with yield, which can be used as the primary index for screening high-efficiency cotton nitrogen. Since the relative nitrogen content, relative nitrogen uptake and relative dry weight showed a very significant negative correlation and a very significant positive correlation, it is recommended as an auxiliary index for screening of nitrogen-efficient varieties.

### Research advances on molecular markers of important traits of cotton stress resistance

Molecular markers of important characteristic genes of cotton mainly concentrated on the screening of molecular markers such as yield and its constitutive factors, fiber quality traits, resistance to rot and wilt. There is less research on abiotic stress resistance, mainly focusing on QTL screening of drought resistance and salinity resistance traits.

#### Screening of molecular markers related to drought resistance of cotton

The high and low permeability of leaf pores is an indication of drought resistance crops. Mauricio et al. (2000) screened the high pore conductivity resource materials by disproportionation selection, and then configured the NM24016/TM-1 separation group to screen two QTL for blade pore conductivity (Wu et al., 2009; Li et al., 2021) (Zhongmian Institute #. 35 × Junmian #. 1) F_2_ to isolate the population. QTL screening for drought resistance was performed by observing the water loss capacity of leaves of cotton after 6 h of natural water loss. A QTL site was discovered on chromosome #16 that controls leaf water loss. The additive effect can explain the phenotypic variation of 12.2% [Lu Mian 97−8 × (12)] F_2_ isolated groups, and obtained data on the impact of constraints on cotton seed yield and plant height in each reproductive period by detecting the restraint index and coefficient, and then screened 7 plant heights in term of site drought resistance, with a contribution rate of 4.5%, 19–19.66%, including 4 plant heights QTL in bud phase, 1 plant height in flowering stage, and 2 plant heights in growth phase. Plant heights of different periods were distributed to different linkage groups, indicating that the same traits are controlled by different loci genes, and plant heights of different reproductive stages are controlled by different genes (Muhammad et al., 2011; Guo et al., 2021), used (FH one 901 × RH one 510) F to separate the group, and detected 7 drought-resistant related QTL. Three QTL were detected under drought conditions, one each for permeability potential, permeability adaptation and plant height, and2 QTL were detected after rehydration. The production of these QTL has laid the ground work for further use of drought-resistant molecular marker-assisted selection techniques and for creation of new drought-resistant strains.

#### Screening of molecular markers related to salt tolerance in cotton

Zhao et al. (2016) isolated the population with (Lu Yan 97−8 × Su 12) F_2_: and detected 4 QTL loci related to salt resistance, contributing 5.47–17.20%, 1 at middle seedling stage and 3 in bud stage (Xu et al., 2021) used two salt-tolerant materials (Zhongo 7 and Zhongmiansuo 35) and two salt-sensitive materials (Xinyan 96-48 and Zhongmiansuo 12) to screen the polymorphism of SSR molecules. These primers are expected to be candidate primers for molecular identification of salt tolerance of cotton (Yao et al., 2011; Zafar et al., 2022) analyzed the salt tolerance inheritance and gene effect of cotton salt tolerance mutant Shannong 011 and its hybrid offspring, and found that the salt tolerance of the mutant was enhanced by controlled nuclear genes, and their inheritance was dominated by additive action. The above research foundation for further research on salt-tolerant and alkali-tolerant cotton by submarker screening and auxiliary selection, and breeding of salt-tolerant varieties.

### Study on the discovery and transformation of key factors of cotton resistance

The gene’s stress response to stress is mainly manifested in the control of its own genetic material and epigenetic regulation under the influence of DNA methylation, RNA disruption or histone modification. There have been many studies on the inverse correlation of stress resistance non- living cotton and the analysis of its energy, which can be divided into two types: one is the functional factor which can enhance the ability of plants to adapt to adverse environments after induced expression by stress, such as *GaP5CS*, *GaTPS*, and Lea. The other is the transcription factor that regulates the expression of the underlying factor and the signal transduction under stress (Duan et al., 2006; Guo et al., 2010), such as *GhERBL*, *GhERF1, GbCBF1*. To date, more than 40 cotton resistance correlation factors have been isolated and identified (Table 1), and their functional characteristics have been preliminarily defined. Based on cloned endogenous or exogenous resistance-related genes, a batch of new cotton materials related to drought resistance and salt resistance have been successfully obtained using transgenic technology (Table 2) introduced Luomian #. 6 of B. buma DNA into two new salt-resistant cotton lines 91-11 and 91-15 with salt content of 0. The lint yield was 19.1% higher than recipient lumian 6 planted in 51% coastal saline area 7 and 237.8%. Combined the choline dehydrogenase gene *betA* with the mutant acetyl lactate synthesizing enzyme. Genes were introduced into three excellent varieties of cotton, and genetically modified plants and their offspring with significantly improved salt resistance were obtained, creating excellent materials for salt-resistant cotton is transmitted to cotton plants by the method of flowering powder tube guidance. The introduction of this gene increases the potassium accumulation of the stems and leaves of cotton, promotes the growth of cotton plants, and accelerates the nutrient growth rate of cotton in the early stage. However, the transgenic material is also manifested in the delayed reproductive period, and the position of stem node of the pregnant flower bud rises. The phenomenon needs to be further improved and studied drought tolerance measurement of the seedling stage, bud stage and flowering stage show that the introduction of exogenous *ZmPIS* genes improves the drought resistance of (GM) genetically modified cotton at different development periods, and after long-term drought stress during the flowering period, the yield of single seed cotton of GM strains is significantly higher than that of wild-type controls. Compared to the large-scale production and use of insect-resistant genetically modified cotton, the genetic engineering of cotton against abiotic stress has made some progress, however it is still in the experimental and research stage, and there is still some distance to commercial application. Compared with the production requirements, research progress needs to be further accelerated.

**TABLE 1:** Genes related to stress tolerance previously cloned in cotton.

Gene	Gene annotation	Function	References
*Lea*	Protein-rich genes in the late fetal stage	Improve the hydrophilic ability of cells	Baker et al., 1988; Magwanga et al., 2018
*GhNHX*	Na^+^/H^+^ reverse transporter gene	Participate in Na^+^ transportation and reduce the concentration of cytoplasmic Na^+^	Wu et al., 2004
*ChPKI*	Threonic acid/threonic acid kinase protein gene	Participate in salt stress signal transduction pathway	Wang et al., 2015
*Cu/Zn*	Copper bell superoxide dismutase gene	Remove intracellular reactive oxygen species	Imlay and Imlay, 1996; Zhou et al., 2019
*SOD*	Water pore protein gene	Regulate the transportation of moisture in the vacuole membrane	Lantzouni et al., 2020
*GhTIPI*	Ion channel protein gene	Regulate ion transport	Gao et al., 2008
*GhCNGC2*	Key thoracic genes in nitrogen acid synthesis	Maintain cell permeability and promote water absorption	Yao et al., 2011
*GaP5CS*	Key foot genes in the synthesis of sea vegetable sugar	Maintain cell permeability and promote water absorption	Yao et al., 2011
*GaTPS*	Metallothionein gene	Induced by low temperature, early drying, salt and ABA	Xue et al., 2009
*GhMT3a*	H^+^-pyroquinoic acid decorative base net	Induced by high salt environment	Rodriguez-Uribe et al., 2011
*GhVP*	S-dorsomethionine synthesis abdominal gene	Induced by high salt environment	Rodriguez-Uribe et al., 2011
*ChSAMS*	Glycosyltransferase with acetylcholine base, brain base flash	Induced by high salt environment	Yuan et al., 2019
*ChGnT*	Cyclophilic protein	Induced by high salt environment	Chen et al., 2019
*GhCYP1*	9-cis-epoxy radish double oxygenated foot gene	Induced by high salt environment	Tan et al., 2015
*ChNCEDI*	9-cis-epoxy carotenoid double oxygenation purchase gene	Induced by high salt environment	Tan et al., 2015
*ChNCED2*	Disulfide bond isomerase gene	Induced by a low nitrogen environment	Iqbal et al., 2020a
*GhPDI*	Protein-dependent breast stimulating gene	Attracted by the high-salt environment	Peng et al., 2014
*GhCPK5*	Thioredoxin gene	Induced by early dry environment	Li et al., 2019
*ChTrx*	Cytokinin-activated protein kinase gene	Induced by high salt and dry environment	Zhang K. W. et al., 2011
*ChMPK2*	Cycloprotein	Induced by high salt and Pseudomonas	Zhang L. et al., 2011
*ChCypl*	Zinc finger protein gene	Regulate the expression of salt tolerance-related genes	Wan et al., 2022
*CSTZ*	Type B reactive element binding protein gene	Regulate the expression of salt tolerance-related genes	Hu et al., 2008
*GhEREB2*	Type B reactive element binding protein gene	Regulate the expression of salt tolerance-related genes	Hu et al., 2008
*GhEREB3*	DRE-binding transcription factor gene	Regulate the expression of salt tolerance-related genes	Huang and Liu, 2006
*GhDBP3*	DREBI/CBF transcription factors	Induced by low temperature, early drying and NaCl	Huang et al., 2007
*GhDREBIL*	DREB/CBF transcription factors	Regulate the expression of genes related to low temperature and salt resistance	Shan et al., 2007
*ChDREBI*	Homologous heterogeneous domain-leucine zipper protein gene	Regulate the expression of salt tolerance-related genes	Ni et al., 2008
*GhERFI*	Type B response factor gene	Regulate the expression of salt tolerance-related genes	Jin and Liu, 2008
*GhDBP2*	DRE-binding transcription factor gene	Regulate the expression of salt tolerance-related genes	Gao et al., 2009
*GhERF4*	Type B response factor gene	Induced by high salt environment	Guo et al., 2009
*ChDREB*	DRE-binding transcription factor	Regulate the expression of salt tolerance-related genes	Meng et al., 2009
*GhZFPI*	CCCH front finger protein gene	Induced by low temperature, early drying, salt and ABA	Meng et al., 2009
*GhNACl*	NAC transcription factors	Induced by low temperature, early drying, salt and ABA	Meng et al., 2009
*GhNAC2*	NAC transcription factors	Induced by low temperature, drought, salt and ABA	Meng et al., 2009
*GhNAC3*	NAC transcription factors	Induced by low temperature, drought, salt and ABA	Meng et al., 2009
*GhNAC4*	NAC transcription factors	Induced by low temperature, early drying, salt and ABA	Meng et al., 2009
*GhNAC5*	NAC transcription factors	Induced by low temperature, drought, salt and ABA	Meng et al., 2009
*GhNAC6*	NAC transcription factors	Regulate the expression of salt tolerance-related genes	Dong et al., 2010
*GhDBPI*	DRE-binding transcription factor gene	Induced by early drying, salt and ABA	Li et al., 2010
*GhDi19-1*	Cys2/His2 zinc finger protein gene	Induced by early dryness, pull and ABA	Li et al., 2010
*ChDi19-2*	Cys2/His2 zinc finger protein gene	Induced by high salt environment	Gao et al., 2009
*ChPTAC*	Plastid transcriptional active factor gene	Induced by low temperature, early drying, salt and ABA	Jin and Liu, 2008
*GhDREB*	DRE-binging transcript factor	Induced by high salt environment	Qiao et al., 2008
*GhDREB2b*	DREB transcript factors	Induced by high salt environment	Niu et al., 2006
*GhEREB2*	Ethylene response element	Regulate salt-tolerance genes expression	Duan et al., 2006
*GhEREB3*	Ethylene response element	Regulate salt-tolerance genes expression	Duan et al., 2006
*GhDREBIA/B*	DRE-binging transcript factor	Induced by low temperature and high salt	Wang et al., 2019
*ChDREBIL*	DREB1/CBF transcript factor	Induced by low temperature, drought and NaCl	Huang et al., 2007
*ChDREBI*	DREB1/CBF transcript factor	Regulate low temperature, salt tolerance related genes expression	Shan et al., 2007
*CüCBFI*	DREB1/CBF transcript factor	Induced by low temperature, drought, salt and ABA	Fei et al., 2021
*ChDBP1*	DRE-binging transcript factor gene	Regulate salt-tolerance genes expression	Dong et al., 2010
*ChDBP2*	DRE-binging transcript factor gene	Regulate salt-tolerance genes expression	Huang et al., 2008
*ChDBP3*	DRE-binging transcript factor gene	Regulate salt-tolerance genes expression	Huang and Liu, 2006
*GhHB1*	Homeodomain-leucine zipper protein gene	Regulate salt-tolerance genes expression	Ni et al., 2008
*GhRCHYl*	Zinc finger protein genes	Drought-induced expression	Gu et al., 2019
*GhSAPI*	Zinc finger protein genes	NaCl-induced expression	Guo et al., 2009
*GhZFPI*	CCCH type Zinc finger protein genes	Regulate salt tolerance related genes expression	Ni et al., 2008
*GhDil9-1*	Cys2/His2 type Zinc finger protein genes	Induced by drought,salt and ABA	Li et al., 2010
*GhDil9-2*	Cys2/His2 type Zinc finger protein genes	Induced by drought,salt and ABA	Li et al., 2010
*GhERFI*	Ethylene responsive factor gene	Regulate salt tolerance related genes expression	Shao et al., 2015
*GhERF2*	Ethylene responsive factor gene	Induced by salt, cold and drought	Jin et al., 2010
*GhERF3*	Ethylene responsive factor gene	Induced by salt, cold and drought	Jin et al., 2010
*GhERF4*	Ethylene responsive factor gene	Regulate salt tolerance related genes expression	Jin and Liu, 2008
*GhERF6*	Ethylene responsive factor gene	Induced by salt, cold and drought	Jin et al., 2010
*GbERF*	Ethylene responsive factor gene	Induced by ethylene, ABA, salt, cold and drought	Qin et al., 2004
*ChNAC1*	NAC transcript factor	Induced by low temperature, drought, salt and ABA	Meng et al., 2009
*ChNAC2*	NAC transcript factor	Induced by low temperature, drought, salt and ABA	Meng et al., 2009
*ChNAC3*	NAC transcript factor	Induced by low temperature, drought, salt and ABA	Meng et al., 2009
*ChNAC4*	NAC transcript factor	Induced by low temperature, drought, salt and ABA	Meng et al., 2009
*ChNAC5*	NAC transcript factor	Induced by low temperature, drought, salt and ABA	Meng et al., 2009
*ChNAC16*	NAC transcript factor	Induced by low temperature, drought, salt and ABA	Meng et al., 2009
*ChWRKY4*	WRKY transcription factors	Induced by drought and salt tolerance	Meng et al., 2009
*GhWRKY5*	WRKY transcription factors	Induced by drought	Meng et al., 2009

**TABLE 2:** Transgenic materials associate with stress resistance in cotton.

Gene	Resource	Phenotype of transgenic plants	References
KATI	Arabidopsis	The accumulation of nutrients at the base and leaves of cotton increases, which promotes the growth of cotton plants and accelerates the initial nutrient growth of cotton	Baber et al., 2020
*GF14λ*	Arabidopsis	Improved the early resistance of genetically modified cotton	Yan et al., 2004
*AtNHXL*	Arabidopsis	Genetically modified cotton has a higher photosynthetic efficiency and nitrogen absorption rate than the wild type under the pressure of 200 mmol-^L–1^ NaCl	He C. et al., 2005
*GST*	Tobacco	Although GST is highly expressed, transgenic plants are resistant to high salt isotopes	Light et al., 2005
*AhCMO*	*Atriplex hortensis*	Improved salt resistance of genetically modified cotton	Hao et al., 2019
*beta*	*Escherichia coli*	The early resistance of transgenic cotton has been significantly improved in the fruit, seedling and flowering periods	Lv et al., 2007
*XL. PEBP*	Sasussured involved Kar.et Kir	The plug resistance of 2 copies of transgenic land elm offspring (T2) materials was significantly higher than that of the control materials.	Zhang K. W. et al., 2011
*TsVP*	Hellungiella halophile	It improves the early resistance of transgenic plants and increases the yield of transgenic plants under early dry conditions.	Lu et al., 2014
*AhCMO*	Atriplex hortensis	The accumulation of betaine increased, the activity of POD and SOD increased, and the early resistance was significantly improved.	Yan et al., 2004
*betA+TsVP*	*E. coli* + Thellangiella halophile	Special gene polymerized plants have higher early resistance than single gene plants.	Lit et al., 2009
*ZmPIS*	Zea mays	Improves the early resistance of genetically modified cotton in various developmental periods	Fujimoto et al., 2000
*AnnBjl*	Mustard	Seepage test function, improved salt resistance	Divya et al., 2010
*AVPI*	Arabidopsis	The early salt resistance of genetically modified cotton has been significantly improved, and the fiber yield is 20% higher than that of the wild type. %	Pasapula et al., 2011
*beta*	*E. coli*	Seepage test function, improved salt resistance	Divya et al., 2010
*phyA*	*Aspergilius ficuum* phytase	The phosphorus content of the leaf tissue of transgenic cotton increased significantly in the seedling stage, the present stage, the flower bell stage and the Turi stage compared with the control.	Pasapula et al., 2011
*GhNHXI*	*Gossypium hirsutum* L.	The germination rate, biological freshness and seedling rate of transgenic strains with a salt content of 0.5–1.5% were higher than that of the control.	Zhang K. W. et al., 2011

## Cotton adversity stress transcription factor gene

Transcription factor (TF), also known as trans-acting factors, are a type of protein molecules that can specifically bind to cis-acting elements in the promoter region of eukaryotic genes to ensure that the target gene is expressed at a specific intensity at a specific time and place (Wu et al., 2021). Under the stress condition, the transcription factor associated with stress tolerance can regulate the expression of anti-retroviral motifs and transmit the stress signal, and the signal can be involved in the information transmission can be cascaded so that, the transcription factor can be used to improve the resistance of plants (Wang et al., 2020). In order achieve a more rational effect, cotton from land (*Gossypium Hirsutum* L.) is currently used. Among them, there are about 30 factors of reverse conversion of cotton resistance the energy properties of which have been preliminarily defined (Table 1).

### Ethylene responsive element binding protein transfer factor

Ethylene responsive element binding protein (EREBP) belongs to the AP2/ERBP family of transcription factors and contains the AP2/ERBP domain (Riechmann and Meyerowitz, 1998). The cis-operating elements derived from EREBP transcription factors are different, which can be divided into two large subfamilies, DREB subfamily and the ERF subfamily member: The DREB subfamily can contain dehydration responsive elements/C repeat (DRE/CRT) identify (Saud et al., 2022) ERF sub-family member can identify GCC-box. Therefore, they play a very important role in the process of plant resistance to abiotic stress, and they are also a key regulator of abiotic stress resistance, the more is currently being introduced into adaptation to adversity (Gao et al., 2009). *GhDREB* -gene from cotton into wheat, and functional analysis showed the following: under drought, high salinity and freezing stress, transgenic plants accumulated a large amount of soluble sugar and the content of chlorophyllin increased significantly compared with the control, but the plant phenotype did not change much (Polge and Polge, 2007; Qiao et al., 2008) isolated *GhRF1* gene from cotton and analyzed its function. GhRF1 gene transcription was found to rapidly accumulate under the stress of ethylene, ABA, high salinity, frost damage, and drought. This gene turned out play an important role in cotton stress. In this laboratory, the genes of anti-retroviral transcription factors *PeDREB* and *HhERF* from the psandophyte plant, *A. chinensis* and *populus euphrica* were cloned and the efficient plant expression vector was constructed (Jeong et al., 2010; Yadav et al., 2016). The pollen tube channel method was used to guide human cotton, and the transgenic lines were obtained. After drought and salt stress treatment, it was found that the drought tolerance of transgenic lines was increased, and the comprehensive analysis of various physiological indexes showed that the salt tolerance of transgenic lines was also increased.

### NAC transfer factor

NAC transcription factors are a special type of transcription factors found in plants. Their common feature is the approximately 150 amino acid long NAC domain with a high degree of protection at the N-terminus, while the C-terminus is a highly variable transcriptional regulatory region. The results indicated that NAC transcription factor played an important role in plant resistance to drought and high salt stress through direct or inducible transcription factor and the expression of drought and high salt stress. It was found that rice tolerance to drought, high salinity and low temperature was increased when rice was overgrown in the vegetative growth stage. Xue et al. (2011) showed higher tolerance and yield in triticale under polyethylene glycol induction. Six NAC transcription factors have been isolated from upland cotton for the first time. They were expressed in roots, stems, leaves and fibers under drought, high salinity, cold and ABA stress, and the highest expression was found in leaves (Meng et al., 2009; Wang, 2010). However, due to the long transformation cycle of cotton, most cotton transcription factor genes are only in the stage of functional identification of gene clones *in vitro* and in model organisms.

### Research and prospects

#### Problems in the research of cotton resistance breeding

Although the cultivars with abiotic stress resistance of cotton were selected, their development was slow compared to the research results of high yield, good quality and diseases and insect resistance. There is still a long way to go to meet the commercial need for stress resistant varieties mainly the following aspects of the problem.

(1) Lack of systematic identification and screening of cotton superior resistance to reverse germplasm resources. For a long time, there have been studies on yield, quality and disease and insect resistance in cotton breeding, but less research on non-biotic stress. Although the researchers have performed relevant work on the screening criteria of cotton resistance and identification of germplasm resources with resistance to resistance, the materials come from different sources of cotton, such as local varieties, extended varieties, introduced materials, and distant hybrid materials. Identification criteria and results from different antagonistic/sensory laboratory resources need to be integrated.
(2) Under tagging of deficiency and resistance elements is closely related, which limits the process of culturing new material aided by under tagging. Molecular markers were used to aid in the selection and polymerization of cotton. Research has mainly focused on improving of fiber quality and disease resistance of cotton. So far, i only preliminary studies of QTL mapping related to drought and salt tolerance have been performed in abiotic stresses (Darmanov et al., 2022), but there are still few sub-markers that are closely linked to the target and can be used directly as molecular markers to assist selection.
(3) The lack of an important value of anti-inverse factor. Although Kronn has a large number of inverse correlation factors of cotton resistance, they are mainly some stress response factors and transfer factors, and few of them have any real value. In meantime, the resistance mechanism also needs to be further elucidated. In addition, in the current cloning of cotton resistance to inverse factor, many in the drought resistance, salt resistance, and cold resistance, high temperature resistance, cotton nutrition efficient use of the target factor is not much.
(4) The theory of seed breeding and the method of sub-design must be thoroughly studied. In the past 10 years, the technology of seed breeding and seed breeding has been highly appreciated, and through the technology of gene transformation a batch of cotton materials with a wide cultivars has been created However, there is a need to do more research on the theory and method of organic combination of seed splitting breeding technology and conventional breeding technology, and the fruit of biomass technology should be used in cotton peanut production.

### Prospects of the study on cotton seed resistance

Compared with the progress of research on resistance of rice, corn, wheat and other food crops, research on resistance of cotton is relatively stagnant, and there is still a certain gap in resistance breeding. In order to better use the research results of molecular biology in the practice of cotton resistance breeding as soon as possible, and speed up the process of cotton resistance breeding, future research, will be conducted based on the exploration and evaluation of excellent resources, the main effect of resistance QTL and the cloning of excellent key resistance genes, using MAS technology (marker assisted selection) and transgenic technology to polymerize resistance genes to achieve the analysis of resistance genes. Sub-design and breeding, to breed high-yielding, -quality, multi-resistant, and widely suitable new varieties of cotton (series). It is recommended to focus on the following aspects of work.

(1) The identification and evaluation of resistant gene-type varieties of cotton are carried out systematically. Through extensive collection of cotton resource materials from different sources, resistance biology identification of germplasm resources and analysis of genetic mechanism of target traits are carried out. Complete and clarify the resistance evaluation indicators under the threat of different adversities, combine the evaluation indicators with field trails, systematically and comprehensively identify and assess resistance gene varieties for cotton, and provide excellent resistance germplasm resources and gene sources for crop breeding practices as soon as possible.
(2) A batch of molecular markers of important resistance traits from cotton QTL were screened and used to aid in the selection of target traits. It is difficult to identify the resilience of different materials on large scale using morphological and physiological indicators. Molecular marker technology has been widely used in various studies of cotton to discover molecular markers closely linked to the characteristic target genes of resistance, and use MAS technology to conduct large-scale screening of the characteristic target genes to find marker resources to provide the design and future breeding of related resistance characteristic molecules.
(3) Explore anti-reverse heterogeneity resources and create new anti-reverse materials. Using omics and other techniques and biological information methods, cotton varieties that contain excellent resistance genes are being studied, and resistance-related genes are discovered and cloned. Functional verification of key genes for resistance is obtained, and their mechanism of action is clarified. Make full use of the research into other crop resistances, direct endogenous and exogenous resistance-genes to human cotton receptors, and create new transgenic materials containing excellent resistance genes to provide excellent genetic resources and target materials for resistance breeding.
(4) Strengthening the combination of basic research and seed breeding and strengthening multidisciplinary cooperation. Although a number of resistance related genes and resistance related transcription factors have been discovered, the endogenous or exogenous resistance related genes have been successfully transferred into cotton using genetic engineering, and the transformation materials with a significantly improved phase table have been obtained. However this work is limited to laboratory research and has not yet been applied to breeding practice. So far, the new varieties with improved non biotic resistance have not been used in production. Each step of cotton resistance breeding involves many scientific researches, such as developing new cotton varieties with high yield, quality, multi-resistance as well as high efficiency and utilization of nutrients through comprehensive assessment.

## Author contributions

SS and LW: conceptualization, writing original draft, and writing review and editing. Both authors contributed to the article and approved the submitted version.
